# Supplementary material for: β-Peltoboykinolic Acid from Astilbe rubra Attenuates TGF-β1-Induced Epithelial-to-Mesenchymal Transitions in Lung Alveolar Epithelial Cells
Source: Molecules. 2019 Jul 15;24(14):2573. doi: 10.3390/molecules24142573 (PMC6680586; doi:10.3390/molecules24142573)
Supplement: Supplementary file 1 [file molecules-24-02573-s001.pdf]

Supporting Information

β-peltoboykinolic acid from *Astilbe rubra* attenuates TGF-β1-induced epithelial-to-mesenchymal transitions in lung alveolar epithelial cells

In Jae Bang [1,†], Ha Ryong Kim [2,†], Yukyoung Jeon [1], Mi Ho Jeong [1], Yong Joo Park [1], Jong Hwan Kwak [1], Kyu Hyuck Chung [1,*]

1 School of Pharmacy, Sungkyunkwan University, Suwon 16419, Republic of Korea; injae753@naver.com (I.J. Bang); jeon1112@skku.edu (Y. Jeon); algh8906@naver.com (M.H. Jeong); pyj084@msn.com (Y.J. Park); jhkwak@skku.edu (J.H. Kwak)

2 College of Pharmacy, Daegu Catholic University, Gyeongsan 38430, Republic of Korea; kimhr@cu.ac.kr (H.R. Kim)

* Correspondence: khchung@skku.edu (K.H. Chung)

Received: date; Accepted: date; Published: date

† These authors contributed equally to this work

Materials and Methods

Cell viability assay

The WST-1 assay was conducted to find an appropriate treatment concentration of test samples in A549 cells. The cells were seeded in 96-well plates at a density of 15 × 10^3 cells/well. After culture for 24 h, the cells were treated with samples for 48 h. Ten microliters of WST-1 reagent (Roche Diagnostics, Montclair, NJ, USA) were added to each well, in accordance with the manufacturer's instructions, and the plates were incubated in 5% CO_2 at 37 °C for 30 min. Cell viability was quantified through the measurement of the absorbance at 440 nm and 690 nm by using a VERSAmax microplate reader (Molecular Devices, Sunnyvale, CA, USA).

Identification of β-peltoboykinolic acid

β-peltoboykinolic acid was identified by spectroscopic analysis including 1H-NMR (850 MHz; Bruker AVNACE III HD 850 MHz NMR spectrometer, Ettlingen Germany) and 13C-NMR (100 MHz; Bruker Ascend 400 MHz NMR spectrometer, Ettlingen, Germany).

Results

Figure S1. A549 cells were incubated with 70% ethanol extract of *A. rubra* whole plant (ARE; 2, 8, 31, 125, and 500 μg/mL) for 48 h, and the WST-1 assay was conducted. ARE decreased the viability of A549 cells in a dose-dependent manner; a cell viability of over 80% was observed after treatment at 125 μg/mL (Figure S1A). A549 cells were incubated with the fractional extracts derived from *A. rubra* extract (8, 31, 125, 250 and 500 μg/mL) for 48 h and cell viability was measured by using the WST-1 assay. In both the rhizome and aerial parts, all solvent fractions at a concentration of 100 μg/mL, except CH_2Cl_2 fractions from the extracts of aerial part and rhizome of *A. rubra* (ARADF and ARRDF, respectively), resulted in a cell viability of over 80%. For ARADF and ARRDF, a cell viability of over 80% was obtained at a treatment concentration of 50 μg/mL (Figure S1B).

Figure S4. The treatment concentration of β-peltoboykinolic acid was determined by WST-1 assay. A549 cells were incubated with β-peltoboykinolic acid for 48 h. Because a significant cytotoxicity was observed at more 20 μg/mL of β-peltoboykinolic acid, 10 μg/mL concentration was determined as maximum treatment concentration.

Table 1. Primers used in qRT-PCR

Primer	Sequence
N-cadherin	Forward: 5′-ACAGTGGCCACCTACAAAGG-3′<br>Reverse: 5′-CCGAGATGGGGTTGATAATG-3′
Vimentin	Forward: 5′-GAGAACTTTGCCGTTGAAGC-3′<br>Reverse: 5′-GCTTCCTGTAGGTGGCAATC-3′
E-cadheirn	Forward: 5′-TGCCCAGAAAATGAAAAAGG-3′<br>Reverse: 5′-GTGTATGTGGCAATGCGTTC-3′
CoL1A1	Forward: 5′-GGCAACAGCCGCTTCACCTAC-3′<br>Reverse: 5′-GCGGGAGGACTTGGTGGTTTT-3′
Snail	Forward: 5′-GAGAACTTTGCCGTTGAAGC-3′<br>Reverse: 5′-GCTTCCTGTAGGTGGCAATC-3′
Fibronectin	Forward: 5′-CAGTGGGAGACCTCGAGAAG-3′<br>Reverse: 5′-TCCCTCGGAACATCAGAAAC-3′
GAPDH	Forward: 5′-AGATCATCAGCAATGCAATGCCTCC-3′<br>Reverse: 5′-ATGGCATGGACTGTGGTCAT-3′

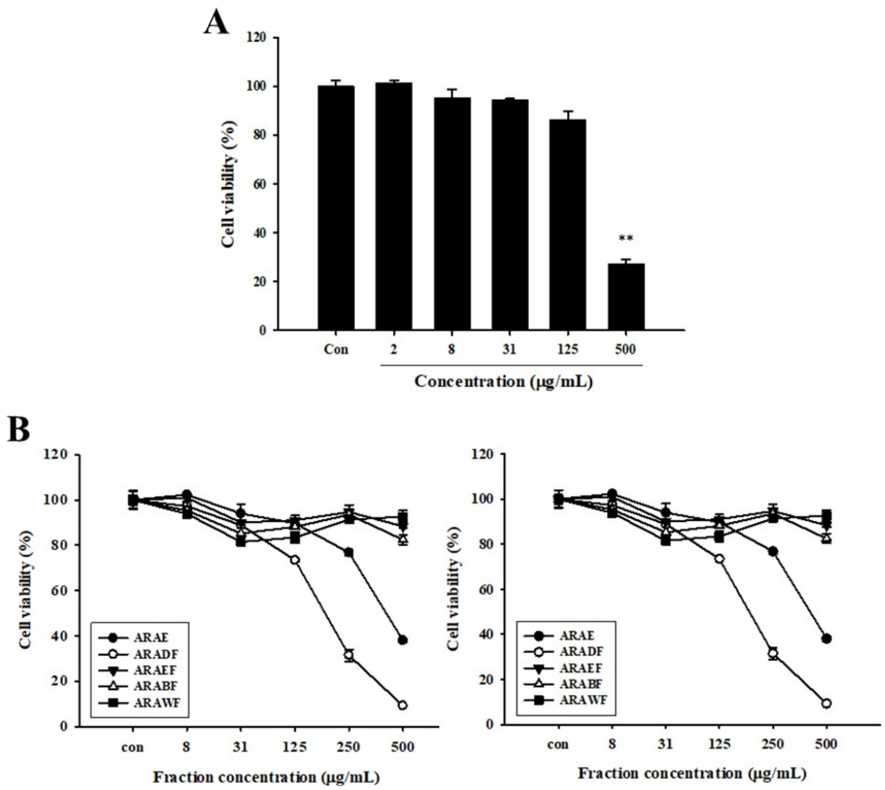

Figure S1. A549 cells were treated with different concentrations of (A) extracts, (B) solvent fractions of *Astilbe rubra* (left, fractions from the aerial part of *A. rubra*; right, fractions from the rhizome of *A. rubra*). Cell viability was evaluated by using the WST-1 assay

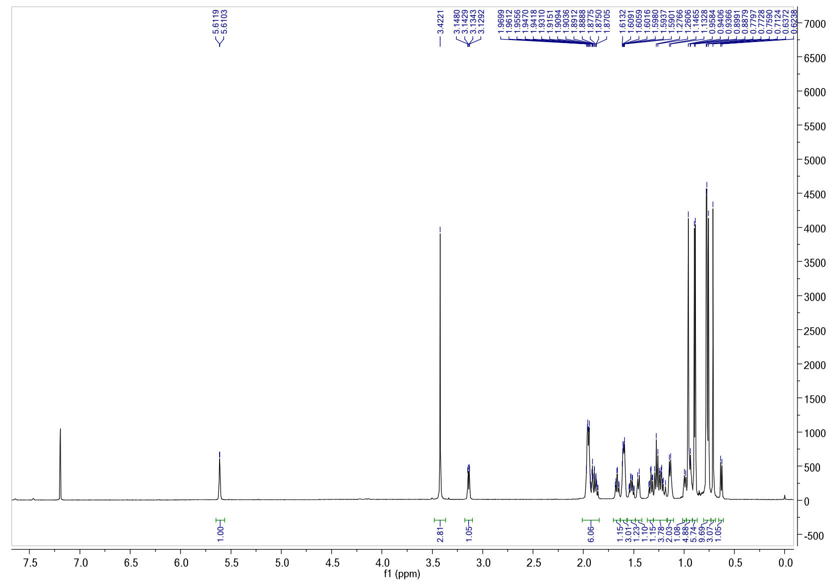

Figure S2A. 1H-NMR spectrum of β-peltoboykinolic acid isolated from Astilbe rubra (CDCl3, 850 MHz).

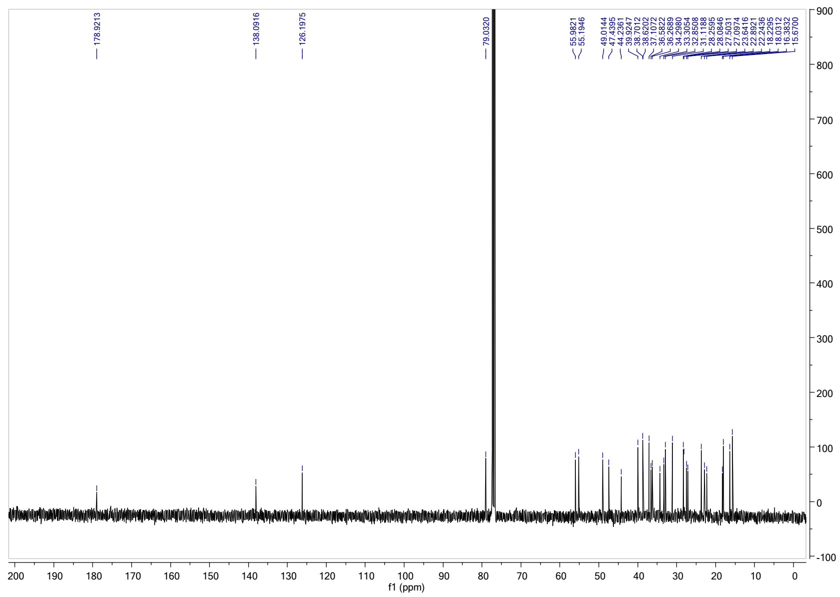

Figure S2B. ^{13}C-NMR spectrum of β-peltoboykinolic acid isolated from *Astilbe rubra* ($CDCl_3$, 100 MHz).

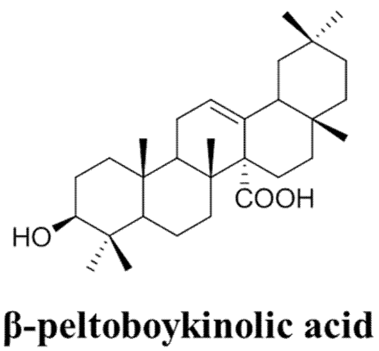

Figure S3. Structure of β-peltoboykinolic acid

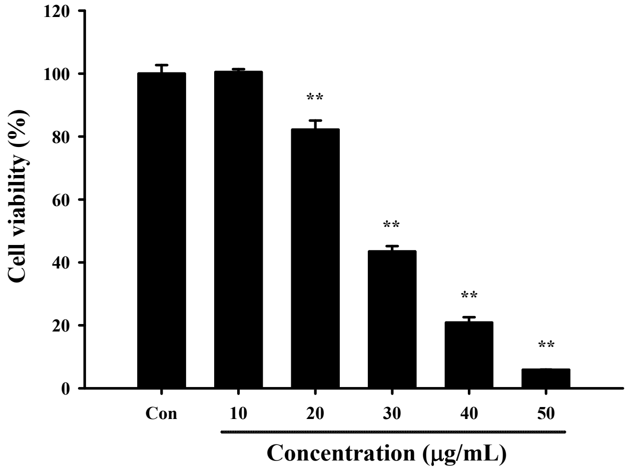

Figure S4. A549 cells were treated with different concentrations of β-peltoboykinolic for 48 h. Cell viability was evaluated by using the WST-1 assay.
